# Mechanical thrombectomy in patients with cervical artery dissection and stroke in the anterior or posterior circulation – a multicenter analysis from the German Stroke Registry

**DOI:** 10.1186/s42466-021-00119-y

**Published:** 2021-05-03

**Authors:** Ludwig Schlemm, Regina von Rennenberg, Eberhard Siebert, Georg Bohner, Fabian Flottmann, Gabor C. Petzold, Götz Thomalla, Matthias Endres, Christian H. Nolte, for the GSR-ET investigators

**Affiliations:** 1 Klinik und Hochschulambulanz für Neurologie, Charité – Universitätsmedizin Berlin, corporate member of Freie Universität Berlin, Humboldt-Universität zu Berlin, Berlin, Germany; 2 Berlin Institute of Health (BIH), Berlin, Germany; 3 Center for Stroke Research Berlin (CSB), Charité – Universitätsmedizin, Berlin, Germany; 4 DZNE (German Center for Neurodegenerative Diseases), Partner Site Berlin, Berlin, Germany; 5 Department of Neuroradiology, Charité - Universitätsmedizin Berlin, corporate member of Freie Universität Berlin, Humboldt-Universität zu, Berlin, Germany; 6 Universitätsklinikum Hamburg-Eppendorf, Klinik und Poliklinik für Neuroradiologische Diagnostik und Intervention, Martinistr 52, 20246 Hamburg, Germany; 7 German Center for Neurodegenerative Diseases (DZNE), Bonn, Bonn, Germany; 8 Department of Neurology, Division of Vascular Neurology, University Hospital Bonn, Bonn, Germany; 9 Universitätsklinikum Hamburg-Eppendorf, Klinik und Poliklinik für Neurologie, Martinistr. 52, 20246 Hamburg, Germany; 10 DZHK (German Center for Cardiovascular Research), Partner Site Berlin, Berlin, Germany; 11 Department of Neurology, Charite – Campus Benjamin Franklin, Hindenburgdamm 30, 12200 Berlin, Germany

**Keywords:** Ischemic stroke, Mechanical thrombectomy, Acute therapy, Dissection, Registry

## Abstract

**Background:**

Cervical artery dissection (CAD) is a rare cause of acute ischemic stroke (AIS) with large vessel occlusion (LVO) and may constitute a challenge for mechanical thrombectomy (MT). We compared procedural characteristics, reperfusion rates, and clinical outcome in AIS patients undergoing MT with and without CAD.

**Methods:**

We performed a pre-specified analysis of patients registered within the German Stroke Registry, a prospectively maintained multicenter registry of consecutive patients with AIS patients treated by MT. Procedural characteristics included time periods and additional application of medication.

**Results:**

Of 2589 patients, 62 (2.4%) were diagnosed with CAD. CAD patients were younger, had lower rates of known vascular risk factors and larger baseline stroke volumes. MT in CAD patients took significantly longer (median [IQR] groin-puncture-to-flow restoration time: 98 [67–136] versus 70 [45–100] minutes; *p* < 0.001) and more often required use of intra-arterial medication (34.4% versus 15.6%; *p* < 0.001). Reperfusion success (modified Treatment in Cerebral Infarction score 2b-3: 85.2% versus 83.3%, *p* = 0.690) and favorable functional outcome after 3 months (modified Rankin Scale score ≤ 2: 70.9% versus 36.4%, adjusted *p* = 0.086) did not differ significantly between patients with and without CAD. The latter findings held true for both CAD in the anterior and posterior circulation.

**Conclusion:**

CAD in AIS requiring MT is rare. MT in patients with CAD constitutes a particular procedural challenge, but still achieves favorable radiological and functional outcomes in most patients. Our data provide indirect evidence that MT is of clinical benefit in patients with AIS due to LVO and CAD.

**Supplementary Information:**

The online version contains supplementary material available at 10.1186/s42466-021-00119-y.

## Introduction

### Background

Mechanical thrombectomy (MT) improves survival and reduces disability in patients with acute ischemic stroke (AIS) due to large vessel occlusion (LVO) of the anterior circulation [1, 2]. The majority of LVO originates from cardioembolic or atherosclerotic etiologies [3–6]. Stroke etiology may modify the endovascular treatment effect [5].

Cervical artery dissection (CAD) represents a rare cause of stroke and is found relatively more often among younger AIS patients and in the aftermaths of minor trauma, such as sport [7–10]. Presence of CAD may impede access to the occlusion site and thereby negatively affect procedural performance metrics although patients with CAD are on average younger and have lower rates of vascular risk factors. Due to these procedural challenges, CAD may be associated with worse outcome. Similar to the current situation with intravenous thrombolysis [11], data on the frequency, safety and efficacy of MT in CAD is relatively scarce. Previous reports have focused on comparisons between use of intravenous thrombolysis in comparison to MT [12]. There are no explicit recommendations on whether or not and how to treat patients with CAD with MT [13].

### Aim

In the current study, we aimed to analyze procedural characteristics and radiological and functional outcome in patients with LVO and CAD who were treated with MT as part of routine care in Germany. Separate subgroup analyses were carried out for patients with anterior and posterior circulation stroke.

## Patients and methods

This is a preplanned analysis insofar as a proposal with a detailed analysis plan had been handed in to the steering committee in July 2017. The proposal was approved by the steering committee of the German Stroke Registry – Endovascular Treatment (GSR-ET) in 2017.

### Data availability

The data that support the findings of this study are available from the corresponding author on reasonable request.

### Study population

For the current study, we analyzed data from patients enrolled in the GSR-ET between April 2016 and April 2018. Details of the registry have been published previously [3, 14]. Briefly, the GSR-ET is an ongoing industry-independent, open-label, academic, prospective, multicenter registry of adult patients (age ≥ 18 years) with a diagnosis of AIS due to LVO treated by MT. Patients were recruited from 12 university hospitals and 13 municipal hospitals from all regions of Germany. All data entered into the registry were assessed locally by trained neuroradiologists and neurologists and underwent centrally coordinated quality checks for consistency, plausibility, and completeness [3, 15].

### Data extraction

The following parameters were extracted from the database: baseline demographic, clinical, and radiological characteristics; procedural characteristics (e.g., number of passages, use of intra-arterial medication, treatment with an extracranial stent); technical outcome (reperfusion success measured by the modified Treatment in Cerebral Infarction [mTICI] scale); functional outcome (National Institutes of Health Stroke Scale [NIHSS] score on day 1 after thrombectomy and on hospital discharge; modified Rankin scale [mRS] score on day 1, at discharge, and on day 90); occurrence of complications; and the presumed etiology of the stroke according to the Trial of Org 10,172 in Acute Stroke Treatment (TOAST) classification [16]. A diagnosis of CAD was made by local investigators based on imaging results and findings from diagnostic tests according to current guidelines. Reperfusion success was defined as mTICI score 2b-3, favorable outcome as mRS score ≤ 2. Intracranial hemorrhage was defined according to the ECASS II (European Cooperative Acute Stroke Study [part II]) definition and refers to symptomatic intracranial hemorrhage [17].

### Statistical analysis

Continuous data are presented as medians and interquartile ranges (IQR), categorical data as percentages. For all parameters of interest, we primarily compared patients with CAD to those without CAD with additional stratification by location of the vessel occlusion (anterior vs. posterior circulation). In addition, we compared patients with CAD and occlusion of a vessel of the anterior circulation to patients with CAD and occlusion of a vessel of the posterior circulation. Baseline parameters, procedural characteristics, and reperfusion rates were compared between groups using Mann-Whitney-U-tests, Pearson chi-squared tests, and univariable ordinal and binary logistic regression analysis. For comparisons of functional outcome parameters (NIHSS score and mRS score), Mann-Whitney tests and univariable regression analyses were used for unadjusted analyses, and analysis of covariance and multivariable regression analyses for adjusted analyses (adjusted for possible confounders that were associated with the grouping variable in univariable analyses and that are known to affect outcome [age, gender, pre-stroke mRS score, medical history, prior antiplatelet and anticoagulation therapy, Alberta Stroke Program Early CT Score (ASPECTS), and location of occluded vessel]). For sensitivity analyses, procedural characteristics and reperfusion rates were also compared with adjustment for potential confounders. A two-sided *P*-value of.05 was considered statistically significant. Due to the exploratory nature of the analysis, no correction for multiple testing was applied.

### Informed consent and ethics approval

Data collection for the GSR was centrally approved by the Ethics Committee of the Ludwig-Maximilians University, Munich (689–15) as the leading ethics committee. Further approval was obtained from local ethics committees or institutional review boards according to local regulations. In accordance with the institutional review board approval, no informed consent was required because no study-specific procedures were performed and data sampling from patients undergoing MT was already mandated by national law for quality control reasons.

## Results

A total of 2589 patients with AIS and LVO treated with MT were included in the current analysis, of whom 62 (2.4%) had a diagnosis of CAD.

### Baseline characteristics

Baseline demographic, clinical, and radiological characteristics of patients with CAD and patients with other stroke etiologies are shown in Table 1. Patients with CAD were significantly younger (median [IQR]; 51 [43–57] years vs. 76 [65–82] years, *P* < .001), less often female (25.8% vs. 50.9%, *P* < .001), and had less pre-stroke disability (*P* < .001). In addition, patients with CAD had lower rates of vascular risk factors and were less often on antiplatelet (17.7% vs. 33.5%, *P* = .009) or anticoagulation medication (1.6% vs. 21.0%, *P* < .001) before stroke. Among patients without CAD, the most common etiology was cardioembolism (51.7%), followed by large-artery atherosclerosis (25.9%) and stroke of undetermined origin (17.5%). Patients with CAD had lower median ASPECTS scores than patients with non-CAD etiologies (8 [6–10] vs. 9 [7–10], *P* = .025), corresponding to larger baseline stroke volumes as detected by CT. Extracranial internal carotid artery stenosis was more common in patients with CAD than in those with other etiologies (46.7% vs. 14.1%, *P* < .001).

**Table 1:** Baseline characteristics

	No CAD, n = 2527	CAD, n = 62	***P***
**Demographic**			
Age – years, median (IQR), n	76 (65–82), n = 2527	51 (43–57), n = 62	<.001
Female, % (n)	50.9% (1286)	25.8% (16)	<.001
Pre-stroke mRS, % (n)			
0	68.0% (1652)	95.2% (59)	<.001
1	12.8% (310)	0% (0)	
2	8.3% (201)	3.2% (2)	
3	6.0% (145)	1.6% (1)	
4	3.5% (86)	0% (0)	
5	1.4% (35)	0% (0)	
Current smoking, % (n)	15.0% (332)	24.1% (13)	.160
**Clinical**			
NIHSS, median (IQR), n	15 (10–19), n = 2527	14 (7–17), n = 62	.107
Medical history, % (n)			
Hypertension	76.8% (1925)	25.8% (16)	<.001
Diabetes mellitus	21.3% (535)	4.8% (3)	<.001
Dyslipidemia	34.1% (854)	17.7% (11)	.006
Atrial fibrillation	41.9% (1049)	1.6% (1)	<.001
Medication, % (n)			
Antiplatelet	33.5% (822)	17.7% (11)	.009
Anticoagulation	21.0% (515)	1.6% (1)	<.001
Etiology, % (n)			
Cardioembolism	51.7% (1307)	–	–
Dissection	–	100.0% (62)	
Large-artery atherosclerosis	25.9% (654)	–	
Small-vessel occlusion	0.0% (1)	–	
Stroke of other determined etiology	4.8% (122)	–	
Stroke of undetermined etiology	17.5% (443)	–	
Direct-to-center, % (n)	52.0% (1313)	54.8% (34)	.701
Symptom onset known, % (n)	61.5% (1553)	74.2% (46)	.047
Time from symptom onset to admission – min, median (IQR), n	127 (59–206), *n* = 1553	119 (54–185), *n* = 46	.728
Time from last-seen-well to admission – min, median (IQR), n	357 (202–685), *n* = 974	283 (200–339), *n* = 16	.163
**Radiological**			
ASPECTS, median (IQR), n	9 (7–10), *n* = 1904	8 (6–10), *n* = 37	.025
Occluded vessel, % (n)			
ICA	24.5% (614)	45.2% (28)	<.001
MCA, M1	56.7% (1423)	29.0% (18)	<.001
MCA, M2	17.7% (444)	19.4% (12)	.420
ACA	2.5% (62)	0.0% (0)	.403
BA	10.2% 255)	19.4% (12)	.032
VA	1.7% (42)	17.7% (11)	<.001
PCA	2.2% (55)	1.6% (1)	1.000
mTICI before thrombectomy, % (n)			
0	85.9% (2088)	86.7% (52)	.801
1	4.8% (117)	5.0% (3)	
2a	2.1% (50)	1.7% (1)	
2b	3.1% (75)	5.0% (3)	
3	4.2% (102)	1.7% (1)	
Extracranial ICA stenosis (> 70%) ipsilateral to intracranial vessel occlusion, % (n/n)	14.1% (334)	46.7% (28)	<.001

*CAD* stands for cervical artery dissection, *IQR* Interquartile range, *mRS* Modified Rankin scale, *NIHSS* National Institutes of Health Stroke Scale, *ASPECTS* Alberta Stroke Program Early CT Score, *ICA* Internal carotid artery, *ACA* Anterior cerebral artery, *BA* Basilar artery, *VA* Vertebral artery, *PCA* Posterior cerebral artery, *mTICI* Modified Treatment in Cerebral Infarction

Initial stroke symptom severity (NIHSS score), smoking status, treatment paradigm (direct-to-center vs. drip-and-ship), delay between time of symptom onset/time last seen well and admission, and mTICI score before thrombectomy did not differ significantly between patients with and without CAD.

Of 28 patients with CAD and ICA occlusion, 5 patients had a concomitant M1 occlusion, 4 patients a concomitant M2 occlusion, and 1 patient a combined M1/M2 occlusion. Of 11 patients with CAD and VA occlusion, 5 patients had a concomitant BA occlusion, and 1 patient had a combined BA/PCA occlusion.

When comparisons were made within the subgroups of patients with stroke either in the anterior or posterior circulation, very similar between-group differences were present in both subgroups. The observed disparities in gender and in the proportion of internal carotid artery stenosis were only present within the anterior circulation subgroup and differences in pre-stroke medication did not reach statistical significance due to smaller sample sizes.

Limiting the comparisons to patients with either anterior or posterior circulation CAD, patients with anterior circulation CAD had significantly shorter median onset-to-admission times than patients with posterior circulation CAD (87 [54–171] min 262 [63–356] min, *P* = .032). The remaining baseline characteristics did not differ significantly between patients with anterior and posterior circulation CAD (Supplemental Table I).

### Procedural characteristics

Intervention data and treatment time metrics are summarized in Table 2. Median time from admission to groin puncture and time from groin puncture to flow restoration was significantly longer in patients with CAD than in patients without CAD (90 [56–126] min vs. 71 [46–104] min, *P* = .019; and 98 [67–136] min vs. 70 [45–100] min, *P* < .001, respectively). Accordingly, total time from symptom onset (if known) to flow restoration was also significantly longer in the group of patients with CAD (322 [233–435] min vs. 245 [190–322] min, *P* < .001). Use of intra-arterial medication was approximately twice as frequent in the CAD group as in the non-CAD group (34.4% vs. 15.6%, *P* < .001).

**Table 2:** Procedural characteristics

	No CAD, n = 2527	CAD, n = 62	***P***
**Intervention**			
Treatment with intravenous thrombolysis, % (n)	55.6% (1393)	67.7% (42)	.069
Anesthesia, % (n)			
Conscious sedation	30.2% (733)	21.0% (13)	.281
Beginning with conscious sedation, switch to general anesthesia	3.7% (90)	4.8% (3)	
Primary general anesthesia	66.1% (1602)	74.2% (46)	
Number of passages, median (IQR), n	2 (1-3), n = 2527	2 (1-3), n = 62	.377
Intra-arterial medication, % (n)	15.6% (387)	34.4% (21)	<.001
Additional heparin bolus given, % (n)	13.3% (303)	27.1% (16)	.006
Concomitant stenting of > 70% ipsilateral extracranial ICA stenosis (if present), % (n)	70.0% (231)	64.3% (18)	.527
**Time intervals**			
Known symptom onset			
Time from symptom onset to flow restoration - min, median (IQR), n	245 (190-322), n = 1553	322 (233-435), n = 46	<.001
Unknown symptom onset			
Time from last-seen-well to flow restoration - min, median (IQR), n	520 (352-833), n = 974	466 (385-524), n = 16	.657
Known and unknown symptom onset			
Time from admission to i.v. thrombolysis - min, median (IQR), n	21 (-76-35), n = 2527	17 (-71-30), n = 62	.518
Time from admission to groin puncture - min, median (IQR), n	71 (46-104), n = 2527	90 (56-126), *n* = 62	.019
Time from groin puncture to flow restoration - min, median (IQR), n	70 (45-100), n = 2527	98 (67-136), n = 62	<.001

*CAD* stands for Cervical artery dissection, *IQR* Interquartile range, *ICA* Internal carotid artery

Within the subgroups of anterior or posterior circulation stroke only, differences in procedural characteristics according to presence or absence of CAD (additional intra-arterial medication, time intervals) were similar to those in the whole population. The only exception was the rate of intravenous thrombolysis, which was significantly higher in patients with CAD in anterior circulation occlusion (80.0% vs. 55.8%, *P* < .001) but not in posterior circulation occlusion (35.3% vs. 49.3%, *P* = .332). Focusing the analysis on patients with CAD only, we found that patients with anterior circulation occlusion were treated with intravenous thrombolysis significantly more often than patients with posterior circulation occlusion (80.0% vs. 35.3%, *P* < .001; Supplemental Table II).

All between-group differences pertaining to procedural characteristics were statistically non-significant in sensitivity analyses considering the differences in baseline characteristics as potential confounders.

### Reperfusion rates

Reperfusion after MT quantified on the mTICI score did not differ significantly between patients with and without CAD (Table 3). No differences were observed within or between the subgroups of patients with anterior or posterior circulation stroke (Supplemental Table III).

**Table 3:** Reperfusion outcome

	No CAD, ***n*** = 2527	CAD, ***n*** = 62	***P***
**mTICI after thrombectomy, % (n)**			
0	9.0% (224)	4.9% (3)	.116
1	1.9% (48)	0.0% (0)	
2a	5.7% (141)	9.8% (6)	
2b	35.6% (881)	54.1% (33)	
3	47.7% (1182)	31.1% (19)	
2b-3	83.3% (2063)	85.2% (52)	.690

*CAD* stands for cervical artery dissection, *mTICI* Modified Treatment in Cerebral Infarction

### Adverse events

Frequency of adverse events did not differ between patients with or without CAD, neither in the whole population nor in the subgroups confined to anterior or posterior circulation stroke. Rates of intracranial hemorrhage, vasospasm and recurrent stroke were below 5% each (Table 4, Supplemental Table IV).

**Table 4:** Adverse events

	No CAD, n = 2527	CAD, n = 62	***P***
Any adverse events, % (n)	13.4% (334)	12.9% (8)	1.000
Device malfunction	0.3% (8)	0.0% (0)	.^a^
Dissection/perforation	2.8% (70)	4.8% (3)	.^a^
Clot migration/embolism	3.1% (78)	4.8% (3)	.^a^
Intracerebral hemorrhage	2.2% (55)	0.0% (0)	.^a^
Vasospasm	2.6% (65)	4.8% (3)	.^a^
New persistent neurological deficit	0.0% (2)	0.0% (0)	.^a^
New transient neurological deficit	0.0% (1)	0.0% (0)	.^a^

^a^ No *P*-value estimated due to low numbers. CAD stands for cervical artery dissection

### Functional outcome

In unadjusted analyses, follow-up NIHSS scores and mRS scores were nominally lower in patients with CAD as compared to patients without CAD indicating better recovery and less severe functional disability. After adjustment for differences in baseline characteristics (younger age and better pre-stroke functional status in CAD patients), these differences were no longer statistically significant. (Table 5, Supplemental Table V). Patients with CAD had similarly high probabilities of a favorable outcome at 90 days irrespective of whether the occlusion was in the anterior or posterior circulation (72.5% vs. 66.7%, *P* = .856).

**Table 5:** Functional outcome

	No CAD, n = 2527	CAD, n = 62	Parameter estimate (95% CI)^**a**^	P_**crude**_ P_**adj**_
**After 24 h**				
NIHSS after 24 h, median (IQR), n	10 (4-20), *n* = 2527	6.5 (2-15), *n* = 62	.^b, c^ -1.39 (-4.85-2.06)^d^	.010^b^ .428^d^
mRS score after 24 h, % (n)				
0	2.8% (60)	10.5% (6)	0.60 (0.13-1.07)^b^ - 0.04 (- 0.71-0.63)^d^	.013^b^ .901^d^
1	6.1% (133)	12.3% (7)		
2	8.7% (190)	7.0% (4)		
3	13.1% (286)	14.0% (8)		
4	18.0% (391)	14.0% (8)		
5	50.5% (1100)	42.1% (24)		
6	0.8% (17)	0.0% (0)		
≤ 2	17.6% (383)	29.8% (17)	-0.69 (-1.27 - -0.11)^b^ 0.35 (- 0.60-1.30)^d^	.020^b^ .472^d^
**At discharge**				
NIHSS at discharge, median (IQR), n	6 (2-13), n = 2527	3 (0-10), n = 62	.^b, c^ 0.23 (-3.05-3.52)^d^	.003^b^ .889^d^
mRS score at discharge, % (n)				
0	8.2% (202)	20.0% (12)	0.95 (0.50-1.40)^b^ 0.26 (-0.34-0.86)^d^	<.001^b^ .403^d^
1	12.0% (296)	21.7% (13)		
2	11.6% (286)	10.0% (6)		
3	13.6% (336)	16.7% (10)		
4	17.0% (420)	10.0% (6)		
5	21.7% (534)	16.7% (10)		
6	15.8% (390)	5.0% (3)		
≤ 2	31.8% (784)	51.7% (31)	-0.83 (-1.34 - -0.32)^b^ 0.20 (-0.55-0.96)^d^	.002^b^ .599^d^
**After 90 days**				
mRS score after 90 days, % (n)				
0	11.7% (260)	18.2% (10)	1.06 (0.59-1.53)^b^ 0.29 (-3.5-0.94)^d^	<.001^b^ .372^d^
1	14.3% (318)	32.7% (18)		
2	10.4% (232)	20.0% (11)		
3	12.4% (275)	5.5% (3)		
4	12.7% (283)	9.1% (5)		
5	10.2% (226)	9.1% (5)		
6	28.4% (632)	5.5% (3)		
≤ 2	36.4% (810)	70.9% (39)	-1.45 (-2.04 - -0.86)^b^ -0.74 (-1.59-0.10)^d^	<.001^b^ .086^d^

^a^Parameter estimates and 95% confidence intervals derived from univariate general linear (NIHSS score), ordinal (mRS distributions), or binary (dichotomized mRS score) regression models; the reference category is formed by patients with CAD. ^b^ Unadjusted models ^c^ No parameter estimate derived from from Mann-Whitney test. ^d^ Analysis adjusted for age, gender, pre-stroke modified Rankin scale score, medical history, prior medication, Alberta Stroke Program Early CT Score, and location of occluded vessel. CAD stands for cervical artery dissection; IQR, interquartile range; mRS, modified Rankin scale; NIHSS, National Institutes of Health Stroke Scale

We performed an additional post-hoc exploratory analysis of the association between treatment with intravenous thrombolysis and outcome in patients with CAD. Adjusting for age, sex, and baseline NIHSS score, we found no significant association for the endpoints NIHSS score at 24 h and at discharge, and mRS ≤ 2 score at 24 h and at discharge (*P* values .707, .530, .252, and .760, respectively). Intravenous thrombolysis was associated with a higher probability of favorable functional outcome at 90 days (31 of 38 [82%] versus 8 of 17 [47%], odds ratio 20.8, 95% confidence interval 2.6 to 168; *P* = .004).

## Discussion

CAD is a rare challenge in MT for AIS with LVO found in 62 of 2589 (2.4%) patients in our large, multicenter registry of academic and non-academic centers mirroring real-world practice in Germany.

With respect to demographics and in line with previous studies, CAD patients in our registry were younger and had lower rates of cardiovascular comorbidities [10]. These characteristics should entail a good prognosis. However, imaging revealed more severe early ischemic changes (as assessed by ASPECTS score) in CAD patients. Lower ASPECTS score may mirror the lack of time to develop cerebral circulation collaterals in time, as arterial dissection is an acute process. Worse collaterals status is independently associated with larger infarct size [18]. Lower ASPECTS may also mirror the higher frequency of ipsilateral carotid stenosis in anterior circulation CAD patients possibly impairing perfusion pressure. Moreover, the median time delay between symptom onset and flow restoration was longer in CAD patients, which is also associated with worse outcome [19]. This longer time delay was at least in part attributable to a longer time period between groin puncture and flow restoration. The latter may reflect that intervention in CAD patients is more demanding, particularly since patients with CAD were younger than patients without CAD and were therefore less likely to have tortuous vessels or high atherosclerotic burden meaning that the CAD itself presumably challenged the interventionalist. The finding that CAD patients received intra-arterial medication more frequently, too, supports the notion of a more demanding intervention in CAD patients.

Nevertheless, despite more severe early ischemic changes on imaging and longer procedural time periods, both radiological (reperfusion) and adjusted functional outcome did not differ significantly between patients with and without CAD. Adverse events did not occur more frequently in CAD patients. The disadvantageous imaging and time parameters may have been balanced by the younger age of CAD patients which is a major indicator for better outcome [20, 21]. Previous data did not appear to be as positive for AIS patients with CAD as found by our real-world data. A post-hoc analysis of the MR CLEAN trial intervention arm and MR CLEAN Registry revealed reperfusion rates that appeared to be lower than those found in our registry (47–58% in MR CLEAN versus 79–84% in our registry) [22]. Other studies examined smaller study populations or did not compare anterior and posterior circulation stroke.

Besides a longer time delay between groin puncture and flow restoration, we also observed a significantly longer delay between admission and groin puncture in patients with CAD. Possible explanations for this observation may include a) less streamlined diagnostic and decision making processes in patients with younger age, i.e. a demographic characteristic not usually associated with a diagnosis of stroke, or b) therapeutic uncertainty once a decision of ischemic stroke due to LVO in the presence of CAD is made.

Additional post-hoc analyses indicated that bridging intravenous thrombolysis may be associated with better functional outcome at 90 days in patients with CAD undergoing MT. While the non-randomized design, the lack of effect in respect to the other functional outcome endpoints, and modest sample size limit the external validity of this observation, it provides reassurance for thrombolytic treatment in this clinical context; further studies are, however, warranted.

To our knowledge, our study is the first to analyze MT in stroke patients with CAD in the posterior circulation separately. Although limited by a relatively small number of patients (*n* = 17), our data provide evidence that support the use of MT for eligible AIS patients with CAD and occlusion within the posterior circulation. In our sample, successful reperfusion (mTICI grade 2b-3) could be achieved in 17/17 patients, adverse events were rare, and most patients (66.6%) were functionally independent after 3 months.

All this should encourage the treating physicians to perform MT in patients with AIS due to LVO in both the anterior as well as the posterior circulation.

We are aware of the following limitations. First, although our data were derived from a large prospective registry, the number of patients with CAD included in the analysis (*N* = 62) was only moderate which could have limited the study’s ability to detect existing between-group differences (type 2 error). Given the low prevalence of CAD among stroke patients in general, collaborative efforts combining data from multiple registries might be helpful in the future. Second, although data checks were conducted at each hospital to ensure complete documentation, we cannot rule out selection bias entirely. Third, management of CAD patients occurred according to current guidelines; however, there was no common protocol for the management of CAD patients executed identically by all hospitals. We therefore do not have detailed data on the order of different maneuvers performed (for example stenting first versus embolectomy first) or the precise peri-interventional antiplatelet regimen used. Registries collecting these information in greater detail will be of benefit for further analyses in the future. Fourth, information about the location of CAD was limited to anterior versus posterior circulation; no information was available regarding the affected vessel or the extent of the dissection. Fifth, we did not assess pc-ASPECTS in posterior circulation occlusion, although pc-ASPECTS would have given a more precise marker of early ischemic changes in these patients. Finally, only presence (or absence) of carotid stenosis was known but not its exact severity in terms of degree, its pathophysiologic origin, and whether or not it was causal to the stroke. However, we assume that the majority of relatively young patients with CAD would not have severe pre-existing atherosclerotic ICA stenosis.

## Conclusion

In conclusion, our analysis demonstrates that AIS patients with LVO due to CAD represent a distinct patient population that poses specific therapeutic challenges for the clinician and the interventionalist. In spite of more severe early ischemic changes on imaging, longer procedure times and higher requirement for concomitant intra-arterial medical treatment, functional outcome does not appear worse than in patients without CAD in both the anterior and posterior circulation. Future studies should aim to identify modifiable contributors to treatment delay in patients with CAD.

## Supplementary Information

**Additional file 1 Supplemental Table I** Baseline characteristics in subgroups of patients with anterior or posterior circulation occlusion. **Supplemental Table II** Procedural characteristics in subgroups of patients with anterior or posterior circulation occlusion. **Supplemental Table III** Reperfusion outcomes in subgroups of patients with anterior or posterior circulation occlusion. **Supplemental Table IV** Adverse events in subgroups of patients with anterior or posterior circulation occlusion. **Supplemental Table V** Functional outcome in subgroups of patients with anterior or posterior circulation occlusion.

## Data Availability

The data that support the findings of this study are available from the corresponding author on reasonable request.
