# Supplementary material for: Mechanical thrombectomy in patients with cervical artery dissection and stroke in the anterior or posterior circulation – a multicenter analysis from the German Stroke Registry
Source: Neurol Res Pract. 2021 May 3;3:20. doi: 10.1186/s42466-021-00119-y (PMC8091690; doi:10.1186/s42466-021-00119-y)
Supplement: Supplementary file 1 — Additional file 1 Supplemental Table I Baseline characteristics in subgroups of patients with anterior or posterior circulation occlusion. Supplemental Table II Procedural characteristics in subgroups of patients with anterior or posterior circulation occlusion. Supplemental Table III Reperfusion outcomes in subgroups of patients with anterior or posterior circulation occlusion. Supplemental Table IV Adverse events in subgroups of patients with anterior or posterior circulation occlusion. Supplemental Table V Functional outcome in subgroups of patients with anterior or posterior circulation occlusion. [file 42466_2021_119_MOESM1_ESM.pdf]

SUPPLEMENTAL MATERIAL

Mechanical Thrombectomy in Patients with Cervical Artery Dissection and Stroke in the Anterior or Posterior Circulation

– A Multicenter Analysis from the German Stroke Registry

Ludwig Schlemm, Regina von Rennenberg, Eberhard Siebert, Georg Bohner, Fabian Flottmann,

Gabor C. Petzold, Götz Thomalla, Matthias Endres, Christian H. Nolte

for the GSR-ET investigators

Supplemental Table I Baseline characteristics in subgroups of patients with anterior or posterior circulation occlusion. 2

Supplemental Table II Procedural characteristics in subgroups of patients with anterior or posterior circulation occlusion. 3

Supplemental Table III Reperfusion outcomes in subgroups of patients with anterior or posterior circulation occlusion. 3

Supplemental table IV Adverse events in subgroups of patients with anterior or posterior circulation occlusion. 4

Supplemental Table V Functional outcome in subgroups of patients with anterior or posterior circulation occlusion. 5

Supplemental Table I Baseline characteristics in subgroups of patients with anterior or posterior circulation occlusion.

	Patients with occlusion in a vessel of the anterior circulation			Patients with occlusion in a vessel of the posterior circulation			CAD-Patients
	**No CAD n=2,207**	**CAD n=45**	**P**	**No CAD n=283**	**CAD n=17**	**P**	**P[a]**
**Demographic**							
Age – years, median (IQR), n	76 (65-82), n=2207	51 (48-56), n=45	.001	75 (65-81), n=283	46 (36-57), n=17	.001	.305
Female, % (n)	52.4% (1157)	20.0% (9)	.001	40.3% (114)	41.2% (7)	1.000	.111
Pre-stroke mRS, % (n)							
0	68.0% (1443)	93.3% (42)		69.0% (189)	100.0% (17)		
1	12.6% (268)	0% (0)		14.2% (39)	0.0% (0)		
2	8.3% (177)	4.4% (2)	.002	6.9% (19)	0.0% (0)	.[b]	.[b]
3	6.2% (131)	2.2% (1)		4.0% (11)	0.0% (0		
4	3.4% (72)	0% (0)		4.4% (12)	0.0% (0)		
5	1.5% (31)	0% (0)		1.5% (4)	0.0% (0/)		
Current smoking, % (n)	15.4% (298)	23.8% (10)	.335	13.0% (32)	25.0% (3)	.301	.635
**Clinical**							
NIHSS, median (IQR)	15 (10-18), n=2,207	14 (7-16), n=45	.100	15 (7-26), n=283	13 (5-26), n=17	.410	.967
Medical history, % (n)							
Hypertension	76.3% (1,672)	26.7% (12)	.001	79.4% (223)	23.5% (4)	.001	1.000
Diabetes mellitus	21.4% (470)	4.4% (2)	.003	20.7% (58)	5.9% (1)	.210	1.000
Dyslipidemia	34.2% (747)	22.2% (10)	.112	33.3% (93)	5.9% (1)	.016	.262
Atrial fibrillation	42.8% (935)	2.2% (1)	.001	34.5% (97)	0.0% (0)	.002	1.000
Medication, % (n)							
Antiplatelet	32.4% (697)	15.6% (7)	.015	41.4% (111)	23.5% (4)	.203	.475
Anticoagulation	21.5% (462)	0.0% (0)	.001	16.8% (45)	5.9% (1)	.325	.274
Etiology, % (n)							
Cardioembolism	53.1% (1,171)	-		41.0% (116)	-		
Dissection	-	100.0% (45)		-	100.0% (17)		
Large-artery atherosclerosis	25.1% (553)	-	-	33.2% (94)	-		-
Small-vessel occlusion	0.0% (1)	-		0.0% (0)	-		
Stroke of other determined etiology	4.3% (96)	-		7.4% (21)	-		
Stroke of undetermined etiology	17.5% (386)	-		18.4% (52)	-		
Direct-to-center, % (n)	52% (1,140)	58% (26)	.454	53.7% (152)	47.1% (8)	.625	.570
Symptom onset known, % (n)	61.8% (1,365)	73.3% (33)	.123	58.0% (164)	76.5% (13)	.203	1.000
Time from symptom onset to admission – min, median (IQR), n	128 (59-203), n=1,365	87 (54-171), n=33	.120	127 (60-243), n=164	262 (63-356), n=13	.177	.032
Time from last-seen-well to admission – min, median (IQR), n	346 (201-676), =842	267 (195-331), n=12	.181	475 (301-780), n=119	348 (270-426), n=4	.422	.485
**Radiological**							
ASPECTS, median (IQR)	9 (7-10), n=1,904	8 (6-10), n=37	.029	-	-	-	- [a]
Occluded vessel, %(n)							
ICA	27.5% (607)	62.2% (28)	.001	-	-	-	-
MCA, M1	63.9% (1,410)	40.0% (18)	.002	-	-	-	-
MCA, M2	20.0% (441)	26.7% (12)	.262	-	-	-	-
ACA	2.8% (61)	0.0% (0)	.633	-	-	-	-
BA	-	-	-	88.3% (250)	70.6% (12)	.049	-
VA	-	-	-	13.4% (38)	64.7% (11)	.001	-
PCA	-	-	-	15.9% (45)	5.9% (1)	.486	-
mTICI before thrombectomy, % (n)	86.6% (1,845)	90.7% (39)		81.5% (221)	76.5% (13)		
0	4.4% (93)	4.7% (2)		7.7% (21)	5.9% (1)		
1	2.1% (44)	2.3% (1)	.416	2.2% (6)	0.0% (0)		.139
2a	3.2% (68)	0.0% (0)		2.2% (6)	17.6% (3)		
2b	3.8% (81)	2.3% (1)		6.3% (17)	0.0% (0)		
3	86.6% (1845)	90.7% (39)		81.5% (221)	76.5% (13)		
Extracranial ICA stenosis (>70%) ipsilateral to intracranial vessel occlusion, % (n/n)	15.7% (327)	61.4% (27)	.001	0.8% (2)	6.3% (1)	.161	.001

[a] P-value for comparing patients with CAD and an occlusion in a vessel of the anterior circulation to patients with CAD and an occlusion in a vessel of the posterior circulation. [b] P value cannot be estimated due to singularities in the Fisher information matrix.
CAD stands for cervical artery dissection; IQR, interquartile range; mRS, modified Rankin scale; NIHSS, National Institutes of Health Stroke Scale; ASPECTS, Alberta Stroke Program Early CT Score; ICA, internal carotid artery; ACA, anterior cerebral artery; BA, basilar artery; VA, vertebral artery; PCA, posterior cerebral artery; mTICI, modified Treatment in Cerebral Infarction.

Supplemental Table II Procedural characteristics in subgroups of patients with anterior or posterior circulation occlusion.

	Patients with occlusion in a vessel of the anterior circulation			Patients with occlusion in a vessel of the posterior circulation			CAD-Patients
	No CAD n=2,207	CAD n=45	P	No CAD n=283	CAD n=17	P	P[a]
**Intervention**							
Treatment with intravenous thrombolysis, % (n)	56% (1231)	80% (36)	.001	49.3% (139)	35.3% (6)	.322	.001
Anesthesia, % (n)							
Conscious sedation	32.8% (696)	28.9% (13)	.555	2.9% (8)	0.0% (0)	.256	.017
Beginning with conscious sedation, switch to general anesthesia	3.8% (80)	6.7% (3)		11.0% (30)	0.0% (0)		
Primary general anesthesia	63.4% (1,346)	64.4% (29)		86.1% (235)	100% (17)		
Number of passages, median (IQR), n	2 (1-3), n=2,207	2 (1-3), n=5	.578	1 (1-2), n=283	3 (1-4), n=17	.171	.797
Intra-arterial medication, % (n)	14.9% (322)	34.1% (15)	.002	22.2% (61)	35.3% (6)	.236	1.000
Additional heparin bolus given, % (n)	13.0% (258)	28.6% (12)	.009	16.1% (42)	23.5% (4)	.496	.759
Stenting of >70% ipsilateral extracranial ICA stenosis (if present), % (n)	69.7% (228)	63.0% (17)	.391	0.0% (0)	100.0% (1)	.333	1.000
**Time intervals**							
Known symptom onset – min, median (IQR), n							
Time from symptom onset to flow restoration	245 (188-320), n=1,365	313 (233-422), n=33	.002	280 (200-350), n=164	395 (233-444), n=13	.203	.566
Unknown symptom onset – min, median (IQR), n							
Time from last-seen-well to flow restoration	512 (350-817), n=842	463 (372-480), n=12	.654	625 (464-949), n=119	568 (568-568), n=4	.881	.500
Known and unknown symptom onset – min, median (IQR), n							
Time from admission to i.v. thrombolysis	21 (-77-34), n= 2207	17 (-67-28), n=45	.757	28 (-75-42), n=283	-34 (-195-58), n=17	.467	.662
Time from admission to groin puncture	70 (46-101), n=2,207	80 (56-114), n=45	.130	78 (53-122), n=283	98 (75-129), n=17	.168	.225
Time from groin puncture to flow restoration	69 (45-97), n=2,207	95 (61-136), n=45	.005	75 (53-113), n=283	101 (82-152), n=17	.051	.382

[a] P-value for comparing patients with CAD and an occlusion in a vessel of the anterior circulation to patients with CAD and an occlusion in a vessel of the posterior circulation. [b] No P-value estimated due to low numbers.
CAD stands for cervical artery dissection; IQR, interquartile range; ICA, internal carotid artery; mTICI, modified Treatment in Cerebral Infarction.

Supplemental Table III Reperfusion outcomes in subgroups of patients with anterior or posterior circulation occlusion.

	All patients			Patients with occlusion in a vessel of the anterior circulation			Patients with occlusion in a vessel of the posterior circulation			CAD-Patients
	No CAD n=2,527	CAD n=62	P	No CAD n=2,207	CAD n=45	P	No CAD n=283	CAD n=17	P	P[a]
mTICI after thrombectomy, % (n)										
0	9.0% (224)	4.9% (3)	.116	8.7% (189)	6.8% (3)	.125	10.2% (28)	0.0% (0)	.207	.449
1	1.9% (48)	0.0% (0)		1.8% (39)	0.0% (0)		2.9% (8)	0.0% (0)		
2a	5.7% (141)	9.8% (6)		5.9% (129)	13.6% (6)		3.3% (9)	0.0% (0)		
2b	35.6% (881)	54.1% (33)		37.3% (809)	47.7% (21)		22.2% (61)	70.6% (12)		
3	47.7% (1182)	31.1% (19)		46.3% (1004)	31.8% (14)		61.5% (169)	29.4% (5)		

[a] P-value for comparing patients with CAD and an occlusion in a vessel of the anterior circulation to patients with CAD and an occlusion in a vessel of the posterior circulation.
CAD stands for cervical artery dissection; mTICI, modified Treatment in Cerebral Infarction.

Supplemental table IV Adverse events in subgroups of patients with anterior or posterior circulation occlusion.

	Patients with occlusion in a vessel of the anterior circulation			Patients with occlusion in a vessel of the posterior circulation			CAD-Patients
	**No CAD n=2,207**	**CAD n=45**	**P**	**No CAD n=283**	**CAD n=17**	**P**	**P[a]**
Any adverse events, % (n)	13.4% (294)	13.3% (6)	1.000	12.5% (35)	11.8% (2)	1.000	1.000
Device malfunction	0.3% (7)	0.0% (0)	.[b]	0.4% (1)	0.0% (0)	.[b]	.[b]
Dissection/perforation	2.8% (61)	4.4% (2)	.[b]	3.2% (9)	5.9% (1)	.[b]	.[b]
Clot migration/embolism	3.2% (70)	6.7% (3)	.[b]	2.1% (6)	0.0% (0)	.[b]	.[b]
Intracerebral hemorrhage	2.3% (51)	0.0% (0)	.[b]	1.4% (4)	0.0% (0)	.[b]	.[b]
Vasospasm	2.8% (62)	4.4% (2)	.[b]	1.1% (3)	5.9% (1)	.[b]	.[b]
Stroke	0.0% (1)	2.2% (1)	.[b]	0.0% (0)	0.0% (0)	.[b]	.[b]
New persistent neurological deficit	0.1% (2)	0.0% (0)	.[b]	0.0% (0)	0.0% (0)	.[b]	.[b]
New transient neurological deficit	0.0% (1)	0.0% (0)	.[b]	0.0% (0)	0.0% (0)	.[b]	.[b]

[a] P-value for comparing patients with CAD and an occlusion in a vessel of the anterior circulation to patients with CAD and an occlusion in a vessel of the posterior circulation. [b] No P-value estimated due to low numbers.
CAD stands for cervical artery dissection.

Supplemental Table V Functional outcome in subgroups of patients with anterior or posterior circulation occlusion.

	**Patients with occlusion in a vessel of the anterior circulation**			**Patients with occlusion in a vessel of the posterior circulation**			**Patients with CAD**
	**No CAD n=2,207**	**CAD n=45**	**P P_{adj}**	**No CAD n=283**	**CAD n=17**	**P P_{adj}**	**P^{a} P_{adj}^{a}**
**After 24 hours**							
NIHSS after 24 hours, median (IQR)	10 (4-19), n=2,207	9 (4-17), n=45	.418<br>.494[b]	9 (3-30), n=283	3 (1-5), n=17	.002<br>.942[b]	.010<br>.969[b]
mRS score after 24 hours, % (n)							
0	2.6% (49)	5.0% (2)		3.8% (9)	23.5% (4)		
1	5.8% (110)	12.5% (5)		9.6% (23)	11.8% (2)		
2	8.7% (166)	10.0% (4)	.032	9.6% (23)	0.0% (0)	.242	.974
3	13.5% (258)	17.5% (7)	.634[b]	10.5% (25)	5.9% (1)	.294	.996[b]
4	18.6% (355)	17.5% (7)		13.0% (31)	5.9% (1)		
5	50.2% (957)	37.5% (15)		51.9% (124)	52.9% (9)		
6	0.6% (12)	0.0% (0/40)		1.7% (4)	0.0% (0)		
**At discharge**							
NIHSS at discharge, median (IQR)	6 (2-13), n=2,207	5 (0-11), n=45	.210<br>.946[b]	5 (1-12), n=283	0 (0-3), n=17	.002<br>.750	.018<br>.812[b]
mRS score at discharge, % (n)							
0	7.9% (170)	11.6% (5)		11.2% (31)	41.2% (7)		
1	12.1% (260)	23.3% (10)		11.9% (33)	17.6% (3)		.054
2	11.9% (255)	9.3% (4)	.008	10.1% (28)	11.8% (2)	.001	.678[b]
3	14.0% (302)	20.9% (9)	.673[b]	10.8% (30)	5.9% (1)	.868	
4	17.3% (371)	14.0% (6)		14.4% (40)	0.0% (0)		
5	21.7% (466)	18.6% (8)		21.3% (59)	11.8% (2)		
6	15.2% (326)	2.3% (1)		20.2% (56)	11.8% (2)		
**After 90 days**							
mRS score after 90 days, % (n/n)							
0	11.7% (228)	17.5% (7)		12.4% (31)	20.0% (3)		
1	14.7% (285)	32.5% (13)		12.4% (31)	33.3% (5)		.901
2	10.4% (203)	22.5% (9)	.001	10.8% (27)	13.3% (2)	.029	.122[b]
3	12.1% (236)	5.0% (2)	.548[b]	12.4% (31)	6.7% (1)	.601	
4	13.4% (260)	10.0% (4)		8.4% (21)	6.7% (1)		
5	9.7% (189)	10.0% (4)		12.8% (32)	6.7% (1)		
6	27.9% (542)	2.5% (1)		30.8% (77)	13.3% (2)		

[a] P-value for comparing patients with CAD and an occlusion in a vessel of the anterior circulation to patients with CAD and an occlusion in a vessel of the posterior circulation. [b] P-value adjusted for age, gender, pre-stroke modified Rankin scale score, medical history, prior medication, Alberta Stroke Program Early CT Score (only anterior circulation occlusions), and location of occluded vessel.

CAD stands for cervical artery dissection; IQR, interquartile range; mRS, modified Rankin scale; NIHSS, National Institutes of Health Stroke Scale.
